# Supplementary material for: Infection-exposure in infancy is associated with reduced allergy-related disease in later childhood in a Ugandan cohort
Source: eLife. 2021 Sep 22;10:e66022. doi: 10.7554/eLife.66022 (PMC8457824; doi:10.7554/eLife.66022)
Supplement: Supplementary file 1. — (a) Model fit statistics for latent class analysis at year 1 and at year 5 considering cumulative infection experience. (b) Results for measurement invariance by sex, at year 1 and at year 5. [file elife-66022-supp1.docx]

**Supplementary file 1a:** Model fit statistics for Latent Class Analysis at year 1 and at year 5 considering cumulative infection experience

|  | **G^2a^** | **AIC^b^** | **BIC^c^** | **CAIC^d^** | **ABIC^e^** | **Entropy** | **DF^f^** |
| --- | --- | --- | --- | --- | --- | --- | --- |
| **Year 1** |  |  |  |  |  |  |  |
| 2-class solution | 126.22 | 160.22 | **256.88** | **273.88** | **202.87** | 0.39 | 238 |
| 3-class solution | 95.27 | **147.27** | 295.11 | 321.11 | 212.51 | 0.56 | 229 |
| 4-class solution | 80.24 | 150.24 | 349.26 | 384.26 | 238.06 | 0.38 | 220 |
| 5-class solution | 70.89 | 158.89 | 409.09 | 453.09 | 269.29 | 0.35 | 211 |
| 6-class solution | 61.69 | 167.69 | 469.08 | 522.08 | 300.69 | 0.46 | 202 |
| 7-class solution | 53.52 | 177.52 | 530.09 | 592.09 | 333.11 | **0.57** | 193 |
| **Year 5** |  |  |  |  |  |  |  |
| 2-class solution | 46.43 | **80.43** | **173.71** | **190.71** | **119.70** | 0.33 | 238 |
| 3-class solution | 29.67 | 81.67 | 224.34 | 250.34 | 141.74 | 0.54 | 229 |
| 4-class solution | 21.85 | 91.85 | 283.9 | 318.90 | 172.71 | 0.54 | 220 |
| 5-class solution | 14.83 | 102.83 | 344.3 | 388.26 | 204.48 | 0.60 | 211 |
| 6-class solution | 10.09 | 116.09 | 406.9 | 459.91 | 238.53 | **0.63** | 202 |
| 7-class solution | 6.45 | 130.45 | 470.7 | 532.66 | 273.69 | 0.59 | 193 |

Bold indicates the best solution model for the corresponding fit index.

^a^G^2^ is the likelihood ratio statistic.

^b^AIC is Akaike Information Criterion.

^c^BIC is Bayesian Information Criterion.

^d^CAIC is consistent AIC.

^e^ABIC is adjusted BIC.

^f^DF is degree of freedom.

**Supplementary file 1b:** Results for measurement invariance by sex, at year 1 and at year 5

|  | **MI^a^** | **G^2b^** | **DF^c^** | **Diff.G^2d^** | **Diff.DF^e^** | ***P*-value** |
| --- | --- | --- | --- | --- | --- | --- |
| Year 1 | Yes | 220.34 | 493 | 17.11 | 16 | 0.379 |
|  | No | 203.23 | 477 |  |  |  |
| Year 5 | Yes | 83.43 | 493 | 14.55 | 16 | 0.558 |
|  | No | 68.88 | 477 |  |  |  |

^a^MI is Measurement invariance

^b^G^2^ is the likelihood ratio statistic.

^c^DF is degrees of freedom.

^d^Diff.G^2^ is the difference of likelihood ratio statistics.

^e^Diff.DF is the difference of degrees of freedom.
